# Lower suicide intention in patients with personality disorders admitted for deliberate self-poisoning than in patients with other diagnoses

**DOI:** 10.1186/s12991-017-0145-3

**Published:** 2017-04-20

**Authors:** T. K. Grimholt, D. Jacobsen, O. R. Haavet, Ø. Ekeberg

**Affiliations:** 1 Department of Acute Medicine, Oslo University Hospital, Nydalen, Pb 4950, Oslo, Norway; 2 Department of General Practice, Institute of Health and Society, University of Oslo, Oslo, Norway; 3 Division of Mental Health and Addiction, Oslo University Hospital, Oslo, Norway; 4 Department of Behavioural Sciences in Medicine, Institute of Basic Medical Sciences, Faculty of Medicine, University of Oslo, Oslo, Norway

**Keywords:** Deliberate self-poisoning, Depression, Hopelessness, Intention

## Abstract

**Background:**

People with deliberate self-poisoning and personality disorders are in increased risk for suicide. Intention and psychiatric features are important factors in a psychiatric evaluation and for planning aftercare.

**Methods:**

Patients admitted to medical departments after deliberate self-poisoning were studied (*n* = 117). Patients with personality disorder according to (ICD-10, F.60-69) were compared to patients with affective disorders, substance use disorders, and unknown psychiatric diagnosis on Beck Suicide Intention Scale (SIS), Beck Suicide Ideation Scale (BSI), Beck Hopelessness Scale (BHS), and Beck Depression Inventory (BDI).

**Results:**

The mean suicide intention score (SIS) was significantly lower among patients with personality disorders compared with patients with other psychiatric diagnoses 10.2 (95% CI 8.1–12.4) vs. 14.6 (95% CI 12.7–16.4) (*p* = 0.040). The hopelessness scores (BHS) were significantly higher among patients with personality disorders 13.0 (95% CI 10.9–15.2) compared with patients with affective disorders 8.2 (95% CI 6.1–10.3) and substance use disorders 9.9 (95% CI 5.2–14.6) (*p* = 0.0014) and unknown psychiatric diagnoses 10.6 (95% CI 9.1–12.2). There were no significant differences between the groups on suicide ideation (BSI) and depression (BDI).

**Conclusions:**

Although patients with personality disorders had lower suicide intention compared to patients with other psychiatric diagnoses, they reported significantly more hopelessness. This distinction is an important implication in the clinical assessment and planning of further treatment of DSP patients.

## Background

Deliberate self-poisoning (DSP) is associated with a high risk of further suicidal behaviour [24] and increased risk of premature death [7]. Patients with personality disorders are at greater risk of repeated suicide attempts [17]. The intention among patients admitted to acute medical wards after an episode of deliberate self-poisoning varies from a “cry for help” up to a serious wish to die [8]. The degree of a wish to die at the time an episode of self-poisoning is associated with higher risk of subsequent suicide [15]. Since higher suicide risk among patients with personality disorders has been demonstrated with an OR of 2.0 (1 95% CI 1.38–2.95) [2], the assessment in the hospital plays a crucial role after an episode of deliberate self-poisoning. Most studies have not measured intention [16]. It is important to provide thorough assessment and a plan for appropriate care before discharge from general hospital. Especially, one of the recognized risk factor for suicide in patients with personality disorders is the less likelihood to ask for, or receive help [19].

The aim was to study suicide intention and psychiatric symptoms, such as hopelessness, suicidal ideation, and depression in patients with personality disorders compared with patients with other psychiatric diagnoses admitted to hospital after an episode of deliberate self-poisoning. We also compared subgroups of patients with affective disorders, substance use disorders, and unknown psychiatric diagnoses.

## Methods

We included patients admitted to acute medical wards in Oslo and Akershus hospital in the period between 2009 and 2014 in accordance with the definition of deliberate self-poisoning [26]. In total, 117 patients were included as a part of baseline data from a multicenter, randomized trial conducted at five hospitals and General Practitioners (GP) in Oslo and Akershus County [13, 14].

A total of 636 patients were assessed for eligibility at the two hospitals Oslo University Hospital and Diakonhjemmet Hospital, whereas 124 were included. The process of inclusion is thoroughly described in the original papers. The patients and the assigning staff were blinded to the treatment category at the time of inclusion to prevent selection bias and baseline data are not biased, because the intervention was carried out after discharge from the hospital.

The inclusion criteria were: adults aged 18–75 years, hospitalized in acute medical wards. Patients with present psychosis, admitted to further psychiatric inpatient treatment, not registered with a GP, with mental retardation or organic cognitive impairment were excluded. Patients that were not able to participate in a clinical interview or fill out a self-report questionnaire because of foreign language were also excluded. Demographic and clinical variables were registered in a form by a study coordinator in each hospital. Patients with personality disorder in accordance with the ICD-10 diagnoses F60-69 were registered. The diagnoses were based on psychiatric assessment and on information from the patients during the assessment plus a review of the medical chart. The groups were analysed based on a classification depending on whether one of the diagnoses was registered in the following order: (1) F60-69 personality; (2) F30-39 affective disorders; (3) F10-19 substance use disorders; and (4) unknown psychiatric diagnosis (in cases of comorbidity where a patient had more than one of the diagnoses, the first in this order was chosen). For the other patients, the diagnoses were less reliable, but most often in the F-40-49 group *adjustment disorder* or *anxiety disorder.* We decided to classify this group as *unknown diagnosis* as we would need a more extensive diagnostic interview to conclude whether a patient had an F 43 diagnosis: reaction to severe stress and adjustment disorders as a reaction to stressful life events or dissociative or somatoform disorders.

We registered gender, age, educational level, and living status. Clinical variables were previous deliberate self-harm, self-poisoning, self-cutting, hospital treatment and received health care before the current episode that leads to hospitalization. In addition, Beck’s scales for intention, suicide ideation, depression, and hopelessness were assessed.

*Beck Suicide Intention Scale* (SIS) is based on a clinical interview of an instrument with 15 items referring to the patient’s precautions and beliefs of the act. Each item is scored on a scale from 0 to 2, with a possible total score of 30 indicating the highest intention of suicide and a wish to die. The questionnaire covers precautions, planning, communication, and expectations regarding the medication load, the degree of planning, and wish to die or live. It is divided into two sections: the first eight items constitute the ‘circumstances’ section (part 1) and are concerned with the objective circumstances of the act of self-harm; the remaining seven items, the ‘self-report’ section (part 2), are based on the patients’ own reconstruction of their feelings and thoughts at the time of the act [6].

*Beck Suicide Ideation Scale (BSI)* is a 19-item instrument that measures the intensity, duration, and specificity of a patient’s thoughts about committing suicide. The scores range from 0 to 38. If the patient scores 0 on both items four and five, which indicates active suicidal desire, the instruction is to skip the next 14 items which address specific suicide plans and attitudes [4].

*Beck Hopelessness Scale (BHS)* is a 20-item scale with true/false statements for measuring positive and negative expectations about the future. The total BHS score ranges from 0 (no hopelessness) to 20 (maximum hopelessness). The classification of scores is: 0–3, minimal; 4–8, mild; 9–14, moderate; and 15–20, severe hopelessness [5].

*Beck Depression Inventory (BDI)* measures the severity of depression during the previous week. It is composed of 21 items related to depressive symptoms. Each item has a set of at least four possible answers, varying in intensity. The standard cut-offs are: scores of 0–9 indicate that a person is not depressed, 10–18 indicates mild-to-moderate depression, 19–29 indicates moderate-to-severe depression, and 30–63 indicates severe depression [3].

### Statistical analyses

Means and frequencies describe demographical and clinical data for the group personality disorders compared with all the other groups combined in Table 1. Chi-square test was used to compare categorical data. Independent sample *t* test and ANOVA were used for normally distributed continuous data to compare groups. To compare all the diagnostic groups on the SIS, BSI, BHS, and BDI, ANOVA was used. To compare the group personality disorders with the other groups combined on the 15 items in the Beck Suicide Intention Questionnaire, the Chi-square test was used. Significance level was set at *p* values <0.05. SPSS vs. 21.0 Chic Il. was used to analyse the data.

**Table 1:** Demographic and clinical variables

(*N* = 117)	F60-69 personality disorder (*n* = 25)	Other diagnoses (F30-39 affective disorders, F10-19 substance use disorders, F40-49 anxiety disorders and unknown psychiatric diagnoses combined)	*p* value
Male	16%	34%	
Female	84%	66%	0.087
Mean age, years (SD)	34.6 (11.6)	40.2 (15.4)	0.099^a^
Living alone	30%	70%	0.111
Educational level			
Elementary school	40%	44%	0.772
College	36%	29%	
Higher education/University	24%	28%	
Previously hospitalized with DSP	29%	71%	0.047*
Contact with health care services because of any DSH *last week* before current episode^b^	65%	36%	0.015*
Previous self-poisoning			
No	12%	37%	0.71
Once	20%	22%	
2–3 times	40%	27%	
4 times or more	28%	14%	
Previous cutting			
No	37%	59%	0.023*
Once	16%	12%	
2–3 times	–	11%	
4 times or more	47%	18%	

The numbers in the table vary from 113 to 117 due to missing responses on single questions

* Significant *p*-value

^a^Independent samples *t* test, the other *p* values are calculated from a Chi-square test

^b^Not all the columns in the Chi-square test are displayed here, only less than 1 week category

### Ethics

The participants were informed at the hospital, received written information, and written consent was obtained in line with the Personal Protection Agency at Oslo University Hospital manual and the Norwegian ethic’s committee that approved the project (ID: S-08708b).

## Results

In total, it was possible to if verify one or more diagnoses in 117 of the 173 included patients; F60-69 personality disorder was registered in 25. The comparison group with other diagnoses consisted of; F30-39 affective disorders (*n* = 35), F10-19 substance use disorders (*n* = 12) and with unknown psychiatric diagnoses (*n* = 45). Demographical and clinical data in the sample are shown in Table 1. There were no significant differences between the patients with personality disorders compared with the group with all the other diagnostic categories combined on the demographic variables. The patients with other or no diagnoses had significantly more often been hospitalized with of deliberate self-poisoning. However, the personality disorder group had been significantly more frequently treated in emergency medical outpatient clinic or with their general practitioner because of deliberate self-harm during the last week before the current episode. There were no significant differences in reported previous episodes of deliberate self-poisoning, but the patients with personality disorders had significantly more often been engaged in self-cutting.

Table 2 shows that the mean score on the Beck Suicide Intention Scale (SIS) was significantly lower in the personality disorder group 10.2 (95% CI 8.1–12.4) compared with the other groups, and highest in the group with unknown psychiatric diagnoses 14.6 (95% CI 12.7–16.4) (*p* = 0.040).

**Table 2:** Suicide intention, suicide ideation, hopelessness, and depression according to diagnostic groups

	F60-69 personality disorders (*n* = 25)	Unknown psychiatric diagnoses (*n* = 45)	F30-39 affective disorders (*n* = 35)	F10-19 substance use disorders (*n* = 12)	
	Mean (95% CI)	Mean (95% CI)	Mean (95% CI)	Mean (95% CI)	*p* value
Beck Suicide Intention Scale^a^	10.2 (8.1–12.4)	14.6 (12.7–16.4)	12.5 (10.1–14.9)	11.3 (6.5–16.1)	0.040*
Beck Suicide Ideation Scale	19.0 (14.6–23.4)	16.1 (12.7–19.5)	16.5 (12.6–20.3)	16.3 (4.0–29.0)	0.680
Beck Hopelessness Scale	13.0 (10.9–15.2)	10.6 (9.1–12.2)	8.2 (6.1–10.3)	9.9 (5.2–14.6)	0.014*
Beck Depression Inventory	27.8 (22.6–33.0)	26.1 (22.4–29.8)	23.0 (20.1–29.0)	21.9 (12.6–31.2)	0.532

ANOVA used to compare all the four diagnoses groups. In this table, the total group used for comparison is split into three subgroups: unknown psychiatric diagnoses, affective disorders, and substance use disorders

* Significant *p*-value

^a^Score ranges from 0 = lowest intention up to 30 = highest intention

There were no significant differences between the groups on suicide ideation and depression; however, the levels were all severe. The hopelessness scores (BHS) were significantly higher among patients with personality disorders 13.0 (95% CI 10.9–15.2) compared with patients with unknown psychiatric diagnoses 10.6 (95% CI 9.1–12.2), affective disorders 8.2 (95% CI 6.1–10.3), and substance use disorders 9.9 (95% CI 5.2–14.6) (*p* = 0.0014) (Table 2).

In the first section of the Beck Suicide Intention Scale related to the circumstances, there was no significant difference [mean score 5.7 (personality disorder) vs. 5.5 (all other psychiatric diagnoses combined), *p* = 0.806]. In the last section related to intentions and expectations about the outcome of the overdose, there was a significant difference [mean score 5.0 (personality disorder) vs. 7.9 (all other psychiatric diagnoses combined), *p* = 0.003].

In Table 3, the comparison on each of the 15 items showed that the personality disorder patients had communicated the impending action more clearly the last year. Their intention was more often a wish to influence others and to a lower degree wanted to die by the poisoning. Furthermore, they did not to the same degree perceive death as a probable outcome of the act or that the ingested substances were lethal.

**Table 3:** Item scores on Beck Suicide Intention Scale according to diagnostic group

	F60-69 personality disorders (*n* = 25)%	Other diagnoses (F30-39 affective disorders, F10-19 substance use disorders, F40-49 anxiety disorders and unknown psychiatric diagnoses combined) (*n* = 86)%	*p* value
Part 1			
Circumstances section			
Isolation			
Someone present	12	22	
Someone nearby	8	29	
Alone	80	49	0.19
Arranged to avoid interference			
Probable	40	42	
Improbable	28	35	
Highly improbable	32	23	0.644
Precautions against being discovered			
None	68	57	
Passive	28	27	
Active (e.g. locked door)	4	16	0.277
Contacted someone to tell			
Contacted someone	71	45	
Contacted but did not tell	13	16	
Did not contact anyone	17	38	0.74
Pre-arrangements for death			
None	72	81	
Thought about it	16	14	
Performed pre-arrangements (will, gave away jewellery etc.)	12	5	0.381
Degree of planning			
None	68	66	
Minimal to moderate	32	29	
Detailed	0	5	0.541
Suicide note			
Did not write	64	63	
Thought about it	8	8	
Wrote note or letter	28	29	0.994
Communicated intention with the act			
None	48	61	
Unclear/indirectly	12	24	
Clearly	40	16	0.026*
Part 2			
Patients’ own reconstruction of their feelings and thoughts			
Intention with the act			
Influence others	24	10.5	
Temporary rest/relief	52	37	
To die	24	52	0.029*
Expected consequences			
Death not probable or did not think about it	44	22	
Death possible	44	40	
Death probable	13	38	0.035*
Perceptions of lethality			
Less than lethal	54	33	
Uncertain	33	32	
Lethal	13	35	0.055
Seriousness of the attempt			
Not serious	30	21	
Uncertain	52	28	
Serious	17	51	0.013*
Ambivalence of living/dying			
Wanted to live	29	20	
Did not care	50	37	
Wanted to die	21	43	0.139
Perceptions of reversibility			
Death improbable if received help	54	33	
Uncertain	13	17	
Certain of dying or did not think about it	33	51	0.164
Degree of intention			
None, impulsive	67	60	
Planned less than 3 h before intake	17	19	
Planned more than 3 h before intake	17	21	0.831

The beck suicide intention interview was not performed for all the patients, and therefore, the numbers are lower in the comparison group

* Significant *p*-value

## Discussion

The main finding was a significantly lower degree of suicide intention in patients with personality disorders compared to all the other diagnoses groups combined and this was especially related to the intention to influence significant others and less expected lethality of the act.

In line with previous research, the patients with personality disorders also reported a significantly more hopeless view of the future [22]. Taken together, these findings are interesting for clinical practice, as both higher intention [15], and level of hopelessness has been demonstrated as predictors for further suicide attempts and subsequent suicide [18, 30].

Furthermore, this distinction is important, because when the clinician assess intention (as is a recommended part of a psychiatric interview in the hospital) and find low intention, this could mask the total picture of the patients state, as the level of hopelessness, and thus, further suicide risk might be underestimated. Hopelessness was predictive of all types of suicidal behaviors in a 13-year follow-up study, where those who expressed hopelessness were 11.2 times as likely to have completed suicide [18].

As demonstrated in previous research, patients with lower levels of suicidal intention received less planned follow-up at the time of discharge from general hospital after self-poisoning [8]. However, the fact that the personality disorder group also had been significantly more frequently in contact with health care services the last week before they were hospitalized is interesting. This may indicate that they presented with suicidal ideation that was not addressed during the recent consultation.

For clinicians, especially in primary care, it is important to be aware if a crisis is emerging and the patient express suicidal ideation that, although the patient did not intend to die, the self-poisoning might under certain circumstances have a fatal outcome. It is, therefore, important to recognize altered illness behaviour in patients with personality disorders and give advice about, e.g., to avoid use of alcohol or substances of abuse that lower threshold to engage in suicidal behaviour and self-harm. Sher and colleagues found that about 50% of patients with borderline personality disorder had a history of comorbid substance use disorder and thus underpins the importance of being cautious [27]. Soloff et al. found no significant differences in the characteristics of suicide attempts between psychiatric inpatients with borderline personality disorder and those with major depressive episode. However, patients with both disorders had the greatest number of suicide attempts and the highest level of objective planning [28].

Additional psychopathology in the personality disorder group, such as depressive disorders or substance use disorders, could have affected the results in our study. However, due to the current design and the low numbers of patients included in this study, it was not possible to pursue any further analyses.

The findings in the current study show that the intention with the self-poisoning among patients with personality disorders was significantly different, as this group to a higher extent wanted to influence other persons. This supports previous research of this population, where interpersonal problems have been linked to suicidal behaviour [31].

Patients with borderline personality disorders and a history of suicide attempt have been described as more aggressive and affectively dysregulated compared with non-attempters [27].

According to the DSM-IV criteria [1], some of the essential features in borderline personality disorders are the impairments in personality functioning and presence of maladaptive personality traits such as neuroticism and easily prone to impulsivity, depression, and anxiety. Frequent feelings of hopelessness and a pessimistic view of the future together with suicide ideation and behaviour is common. In the current study, the levels of hopelessness were significantly higher, while suicide ideation and depression were not significantly different but higher. However, although there are differences in phenomenology, longitudinal course among, e.g., bipolar disorders and borderline personality disorders, and the findings of comorbidity studies are equivocal [25], there is a need for further research into this in the current population.

Furthermore, because patients with personality disorders can exhibit a pattern of more rapid shifts in affect related to environmental events, in contrast to depressive disorders, it would have been interesting to further investigate whether there are differences in eventual changes of psychiatric symptoms across the diagnostic groups over time after discharge from the hospital.

As demonstrated by Lawn, patients with personality disorders found it challenging to seek help from hospital emergency departments during crises [19]. In the current study, there were significantly fewer patients with personality disorders that had been previously hospitalized with self-poisoning (29 vs. 71%), although the numbers of self-reported non-hospitalized self-poisoning were higher. These results underpin Lawns findings and need to be further investigated. However, it could also indicate that although the reported frequency of previous self-harm was higher, the seriousness and lethality were lower and, therefore, could be treated at a lower level of health care, possibly without impairing treatment quality, in line with the policy in the Norwegian health care system [21].

Nevertheless, it is important to use the opportunity to provide sufficient follow-up at the time of discharge from hospital. Although evidence of effective treatment after deliberate self-harm from clinical trials is sparse in general, findings in a recent Cochrane review support a substantial role for psychotherapy in the treatment of people with borderline personality disorder [29].

In two studies of patients admitted to emergency departments after a suicide attempt, the mean Beck hopelessness score was 9.6 and 10.2, respectively [9, 11]. In a similar Swedish study, the mean scores of the Beck Hopelessness Scale for the total group were 10.4. For the diagnostic groups, the scores were 9.3 for patients with substance use disorders, 9.0 for depressive disorders, and lowest for the adjustment disorders 7.5 [23]. Lester, Beck, and Steer studied patients admitted to hospital for suicide attempts and found no differences on the depression inventory scores when they compared the depressive attempters with patients that described illicit activities or diagnosed with anti-social, drug, or alcohol personality disorders [20]. In concordance with our findings, the latter group also reported lower suicide intent than those diagnosed with depression, although there were no significant differences between the diagnostic groups on the depression inventory in our study.

## Strengths and limitations

There are some limitations in this paper. The reliability of the personality disorder diagnoses would probably have been improved, and particularly if a structured interview had been used. All patients had a psychiatric assessment, and for most of them, there were access to records from previous hospitalizations. In addition, only major diagnostic groups were classified, which strengthens the validity. In addition, the diagnoses were like in similar studies [10, 12] registered from the patient’s chart. Furthermore, it is more likely that the number of patients with personality disorder in the current sample is underreported rather than the other way, as the diagnosis was based on records from previous psychiatric and medical treatment. The frequency of personality disorders among deliberate self-poisoning was also similar to a comparable study, where clinicians found that 22.6% had a borderline personality disorder [10]. In a clinical setting, the assessing personnel will mainly have information available from the patients themselves and the medical records, and thus, our finding resembles the clinical practice. Second, this method did not enable us to analyse any distinction between patients with borderline personality disorders and the other forms of personality disorder, as the first group in particular is known to have increased suicidal risk [16]. Third, our findings must be interpreted in the context of a somatic hospital setting and the severity of psychiatric symptoms found in other studies and the lethality of the overdose may differ from patients seen in, e.g., primary care out patient settings not requiring medical treatment and or patients treated in psychiatric inpatient care. It should also be noted that we due to the study design excluded the patients admitted to further psychiatric inpatient treatment.

Finally, people with or without personality disorder, which attempt suicide solely treated in primary care, is a possible confounding factor.

The strengths of this paper are that these findings to our knowledge have not previously been addressed, and are relevant for clinicians that treat a high number of deliberate self-poisoning patients in the hospitals. Furthermore, the high numbers included in each group make the comparisons in the statistical analysis more robust and thus the external validity and generalizability of the results in spite of the combination of the other or no diagnoses into one group. Finally, the use of validated scales strengthens the reliability of the results.

## Conclusion

Patients with personality disorders reported significantly lower suicide intention compared to patients with affective, substance use disorders, unknown psychiatric diagnoses. This was mainly due to the expected outcome from the poisoning, as the personality disorder patients more often indented to influence others, and did not expect that the overdose was lethal. The patients with personality disorders also reported significantly more hopelessness, but not significant different levels of depression and suicide ideation. Taken together, this underlines the importance of carrying out a thorough assessment in the hospital and not only emphasizes suicidal intention when planning for aftercare.
